# Trapping toxins within lipid droplets is a resistance mechanism in fungi

**DOI:** 10.1038/srep15133

**Published:** 2015-10-14

**Authors:** Wenqiang Chang, Ming Zhang, Sha Zheng, Ying Li, Xiaobin Li, Wei Li, Gang Li, Zhaomin Lin, Zhiyu Xie, Zuntian Zhao, Hongxiang Lou

**Affiliations:** 1 Department of Natural Product Chemistry, Key Lab of Chemical Biology of Ministry of Education, Shandong University, No. 44 West Wenhua Road, Jinan City, Shandong Province, China; 2 College of Life Sciences, Shandong Normal University, No. 88 East Wenhua Road, Jinan City, Shandong Province, China

## Abstract

Lipid droplets (LDs) act as intracellular storage organelles in most types of cells and are principally involved in energy homeostasis and lipid metabolism. However, the role of LDs in resistance to toxins in fungi remains largely unknown. Here, we show that the trapping of endogenous toxins by LDs is a self-resistance mechanism in the toxin producer, while absorbing external lipophilic toxins is a resistance mechanism in the toxin recipient that acts to quench the production of reactive oxygen species. We found that an endolichenic fungus that generates phototoxic perylenequinones (PQs) trapped the PQs inside LDs. Using a model that incorporates the fungicidal action of hypocrellin A (HA), a PQ derivative, we showed that yeast cells escaped killing by trapping toxins inside LDs. Furthermore, LD-deficient mutants were hypersusceptible to HA-mediated phototoxins and other fungicides. Our study identified a previously unrecognised function of LDs in fungi that has implications for our understanding of environmental adaptation strategies for fungi and antifungal drug discovery.

Microorganisms have evolved diverse strategies to adapt to various environmental stresses^1^,^2^,^3^. Self-resistance in a toxin-producing organism and toxin-resistance in the recipients are some of the most important adaptive abilities. The emergence of resistance to endogenous or extracellular toxins results via one or a combination of the following mechanisms: (i) intracellular modification or degradation of toxins^4^,^5^,^6^,^7^,^8^,^9^, (ii) target mutation^10^,^11^, and (iii) removal of the toxins by efficient efflux pumps^12^. Numerous studies have shown that subcellular organelles are also involved in resistance to oxidative stress and intracellular toxins. For example, chloroplasts, mitochondria and peroxisomes, the major generators of intracellular reactive oxygen species (ROS), harbour antioxidant defence systems that confer resistance to endogenous oxidative stress^8^. Lysosomes in mammalian cells and vacuoles in yeast are known to degrade molecules, such as nutrients or metabolites, that have essential functions at physiological concentrations but that become toxic in excess^13^,^14^,^15^.

Lipid droplets (LDs) are dynamic intracellular organelles that contain neutral lipids, including triacylglycerols (TAGs) and sterol esters (SEs), as their main constituents. These are surrounded by a phospholipid monolayer and specific proteins, and they are ubiquitously present in eukaryotic and prokaryotic cells^16^,^17^. LDs have been assumed to be inert fat particles used to store carbon and energy since they were first recognised in the 19^th^ century^18^,^19^,^20^. Recent evidence has shown that LDs participate in multiple cellular functions, such as membrane trafficking, phospholipid recycling, intracellular protein metabolism and cell signalling^20^,^21^,^22^. Macrophages utilise LDs to store esterified cholesterol when excess endogenous cholesterol causes endoplasmic reticulum (ER) stress^23^. In response to starvation, autophagosomes can mediate the delivery of LD content into lysosomes for degradation^24^. Notwithstanding the important cellular functions of LDs, little is known about the involvement of LD-mediated resistance in environmental adaptation.

Here, we show that phototoxic perylenequinones (PQs), which are biosynthesised by the endolichenic fungus *Phaeosphaeria* sp., are taken up into LDs following exposure to light irradiation. This confers resistance in the producer to these phototoxins. Using a PQ derivative, hypocrellin A (HA), as a molecular probe, we found that trapping HA inside LDs resulted in reduced phototoxicity against both the pathogenic fungus *Candida albicans* and the yeast model *Saccharomyces cerevisiae*, which suggests a function for this exquisite organelle in drug resistance. In addition, mutants deficient in LDs were found to be more hypersusceptible to HA-mediated phototoxins and other fungicidal agents. We believe that this LD-mediated resistance mechanism may provide valuable insights into how self-resistance to endogenous toxins is conferred to toxin producers and how resistance to external toxins occurs in other recipients.

## Results

### Trapping phototoxins in LDs serves as a resistance mechanism in an endolichenic fungus

**Figure 1:**
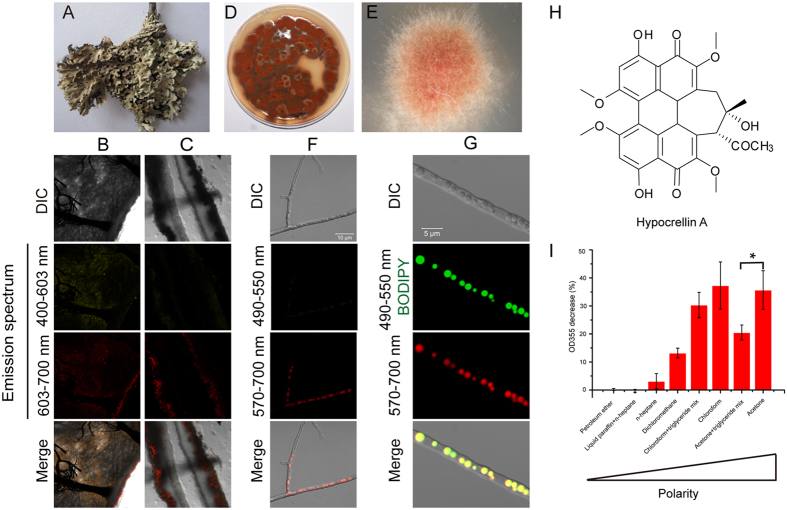
Trapping phototoxins in lipid droplets probably serves as a resistance mechanism in an endolichenic fungus. (**A**) An image of the thallus of lichen, *Heterodermia obscurata* (Nyl.) Trevis. (**B**,**C**) CLSM observation of a thallus cut transversely (**B**) or longitudinally (**C**). (**D**,**E**) Photomicrographs of the isolated endolichenic fungus colony. (**F**) Filaments of isolated fungi, observed by CLSM. The fluorescent substances produced by filaments were excited by 488 nm and 555 nm lasers and observed at the two indicated emission spectrums. The fluorescent substances within the filaments displayed red fluorescence in the emission spectrum of 570–700 nm rather than 490–550 nm. (**G**) Intracellular distribution of fluorescent substances. The filaments were stained with BODIPY, a dye that is specific for LDs, and observed by CLSM using the same conditions described in panel (**F)**. Emission spectrums of 490–550 nm and 570–700 nm were used to detect BODIPY staining and fluorescent agents, respectively. (**H**) The chemical structure of HA. (**I**) Detection of singlet oxygen molecules produced by HA under light irradiation in different solvents. Data represent mean values ± SD from three independent replicates.

Our previous investigation of an endolichenic fungus, *Phaeosphaeria* sp., from the lichen *Heterodermia obscurata* (Nyl.) Trevis (Fig. 1A–E), led to the discovery of a series of PQs that harbour cytotoxic activity due to the high yields of reactive oxygen species (ROS) they produce upon light irradiation^25^,^26^. We sought to understand the self-resistance mechanisms that this producer evolved to these phototoxins.

The perylenequinonoid skeleton of PQs makes fluorescent microscopic analysis a convenient method by which to study these compounds. Examination by confocal laser scanning microscopy (CLSM) showed that the thallus of the lichen was composed of filaments and algae cells (Fig. 1B,C). Stereoscopic analysis using z-stacks demonstrated that the filaments were embedded within the lichen (Supplementary Results and Supplementary Video 1). The filaments produced substances that displayed orange and red fluorescence (Supplementary Fig. 1 and Supplementary Video 1), in agreement with the characteristics of isolated PQs^25^. The endolichenic fungus *Phaeosphaeria* sp., which produces the phototoxins, was isolated and purified (Fig. 1D,E). When the fungus was exposed to light, it grew at a much slower rate compared with that cultured in darkness (Supplementary Fig. 2A). We observed that pigments accumulated in the LDs of the fungi filaments, as indicated by BODIPY staining of the LDs (Fig. 1F,G). By contrast, red fluorescent granules were secreted by the dark-cultured organisms (white arrow, Supplementary Fig. 2B). Because PQs can be activated by light to generate high quantum yields of ROS, especially singlet oxygen (^1^O_2_), which results in phototoxicity^25^,^26^,^27^, the ability of the microorganism to trap PQs in LDs led us to hypothesise that LDs may play a key role in self-resistance.

To test this hypothesis, we used the PQ derivative HA (Fig. 1H), which is the main constituent in *Phaeosphaeria* sp. and displays potent antifungal, antibacterial and antivirus activities^25^,^27^,^28^,^29^,^30^, as a molecular tool for an *in vitro* study. Because it is a hydrophobic agent, HA has a very high octanol-water partition coefficient (*K*_OW_) with a Log P greater than 4, and it is easily dissolved in lipophilic solvents. When HA was dissolved in different solvents of variable polarity or in solvents containing triglycerides to mimic LD components, light-driven ^1^O_2_ production by HA was proportional to the polarity of the solvents (Fig. 1I). The lower ROS production observed when HA was in nonpolar solvents suggested that their localisation in LDs may be a resistance mechanism in this endolichenic fungus.

To explore whether this specific mechanism is also used by other fungi, further experiments were performed to evaluate the effect of LD size or LD formation on resistance to the phototoxicity of HA.

### Large LDs confer resistance to lipophilic toxins in yeast

**Figure 2:**
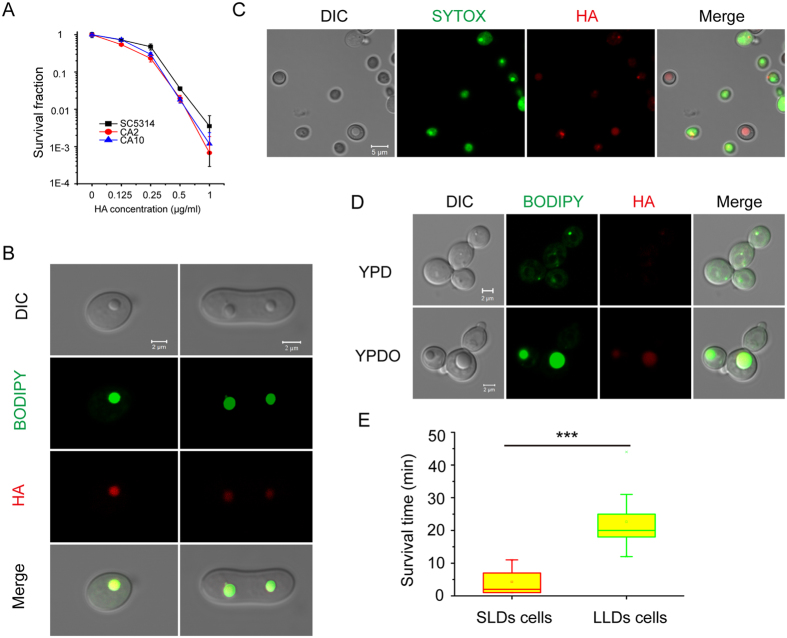
*C. albicans* cells with large LDs are resistant to the phototoxicity of HA. (**A**) The killing curve of HA-mediated photodynamic inactivation against *C. albicans* cultured in YPD medium. *C. albicans* SC5314, CA2, CA10 strains were cultured in YPD medium. Cells grown overnight were diluted to 10^6^ CFUs/ml and exposed to HA with light irradiation for 10 min. The surviving cells were then measured by the colony counting method. (**B**) The distribution of HA in *C. albicans* cells. *C. albicans* SC5314 cells were cultured in SD medium and stained with BODIPY to visualise LD localisation. Prestained cells were incubated with HA for CLSM observation. (**C**) The viability of *C. albicans* cells that were treated with HA-mediated photodynamic inactivation. *C. albicans* SC5314 cells cultured in YPD medium were treated with HA and exposed to light irradiation. After 10 min, cells were stained with the live/dead-indicating dye SYTOX for CLSM observation. (**D**) The amounts of HA trapped in LDs of *C. albicans* that were cultured in YPD or YPDO medium*. C. albicans* SC5314 cells were cultured in YPD medium or YPDO medium. After overnight growth, cells were prestained with BODIPY and incubated with HA for CLSM observation. (**E**) Comparison of survival times of *C. albicans* cells between cells cultured in YPD medium with small LDs (SLDs) and cells cultured in YPDO medium with large LDs (LLDs) (n = 29 for SLDs cells, n = 36 for LLDs cells). One-way ANOVA and Bonferroni test were used to evaluate the difference in survival times. ***p < 0.001.

To determine the relationship between LD size and resistance to external toxins, HA was again used to assess light-induced toxicity in the pathogenic fungus *C. albicans.* The majority of *C. albicans* strain SC5314 cells were rapidly killed by HA when exposed to light irradiation, with few viable sub-populations remaining (Fig. 2A). The in-dark test verified that HA was accumulated in vesicle-like structures identified as LDs by BODIPY staining^31^ (Fig. 2B) rather than in vacuoles or the nucleus, as indicated by CMAC and DAPI staining, respectively^32^,^33^ (Supplementary Fig. 3). Further tests showed that the trapped HA reached its peak concentration within 10 minutes of incubation (Supplementary Fig. 4). Using two other PQ derivatives, phaeosphaerin A (PA) and phaeosphaerin C (PC), as probes (Supplementary Fig. 5) or by changing the host from *C. albicans* to *S. cerevisiae* (Supplementary Fig. 6), we observed that the phototoxic PQs were trapped in the LDs of the fungal cells. CLSM examination showed that the surviving yeast cells were phenotypic variants with large LDs (Fig. 2C).

To further demonstrate that an increase in the size of the LDs confers resistance, oleate was used to increase LD size (Supplementary Fig. 7). We found that larger LDs trapped larger amounts of HA when cells were cultured in YPD medium containing oleate (YPDO), which confirmed the storage function of LDs (Fig. 2D). We then studied the relationship between the size of LDs and cell fate in response to HA-mediated phototoxins in real time. Time-lapse experiments showed that the onset of cell death was greatly delayed in cells with larger LDs (LLDs) that were cultured in YPDO medium compared to cells with small LDs (SLDs) that were cultured in YPD medium (Supplementary Fig. 8 and Videos 2 and 3). Furthermore, statistical analysis of survival times revealed that cells with LLDs were much more resistant to HA-mediated phototoxins than did cells with SLDs (Fig. 2E).

### LD-deficient yeast mutants are much more sensitive to lipophilic toxins

**Figure 3:**
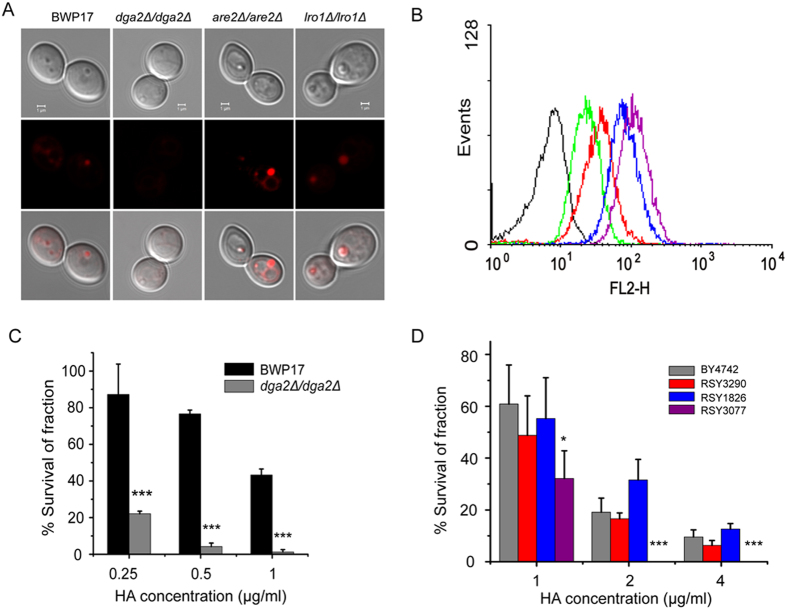
LD-deficient yeast mutants are hypersusceptible to lipophilic toxins. (**A**) The LD morphology of *C. albicans* mutant strains stained with Nile red as revealed by CLSM observation. (**B**) Measurements of LD size in *C. albicans* mutant strains, which were determined by Nile red staining and flow cytometry. Black line, negative control; red line, BWP17; green line, *dga2*Δ*/dga2*Δ; blue line, *are2*Δ*/are2*Δ; and purple line, *lro1*Δ*/lro1*Δ. (**C**) The susceptibility of *C. albicans* LD-deficient mutant strain *dga2*Δ*/dga2*Δ and its parent strain BWP17 to HA-mediated photodynamic inactivation. (**D**) The susceptibility of *S. cerevisiae* mutant strains to HA-mediated phototoxicity. Data represent mean values ± SD from three independent replicates. Student’s *t* test was used to determine significant differences. *p < 0.05, ***p < 0.001.

To validate the important role of LDs in drug resistance, we tested the susceptibility of LD mutant strains to the toxicity of HA. The core lipids—triacylglycerols (TAGs) and sterol esters (SEs)—are known to be the major constituents of LDs^20^. In *S. cerevisiae*, Are1p and Are2p are primarily responsible for the synthesis of SEs, and Dga1p and Lro1p are responsible for the formation of the majority of the TAGs^34^. Based on the homologous genes in *S. cerevisiae*, the genes *ARE2* (orf19.2248), *LRO1* (orf19.6018) and *DGA2* (orf19.6941) in *C. albicans*, which share sequence identity with their *S. cerevisiae* orthologues of 45.54%, 55.00% and 32.99%, respectively, were analysed to determine their roles in LD formation. The mRNA levels of *ARE2*, *DGA1* and *LRO1* were upregulated in response to oleate stimulation, suggesting a possible function for these genes in driving LD formation (Supplementary Fig. 9). We then created the mutant strains *are2*Δ*/are2*, *lro1*Δ*/lro1*Δ and *dga2*Δ*/dga2*Δ via homologous recombination (Supplementary Methods). CLSM observations and flow cytometry measurement revealed that only the *dga2*Δ*/dga2*Δ strain lost the ability to form LDs (Fig. 3A,B), consistent with a recent report that the two isoforms of diacylglycerol acyltransferase are responsible for neutral lipid synthesis in *Candida tropicalis*^35^. We then tested the susceptibility of the mutant strain *dga2*Δ*/dga2*Δ to HA-mediated photodynamic inactivation. The *dga2*Δ*/dga2*Δ strain became more susceptible to HA compared with the parent strain BWP17 when exposed to light irradiation (Fig. 3C).

The LD-deficient mutants of *S. cerevisiae*^31^ were also used to test susceptibility to HA-mediated photodynamic inactivation. A single defect in TAG synthesis (*are1*Δ *are2*Δ) or SE synthesis (*dga1*Δ *lro1*Δ) had a smaller effect on sensitivity to HA-mediated phototoxicity. However, the quadruple mutant strain (*dga1*Δ *lro1* Δ*are1*Δ *are2*Δ), which does not form neutral lipids, was highly susceptible to the photodynamic inactivation of HA (Fig. 3D).

### LDs quench intracellular ROS production caused by HA

**Figure 4:**
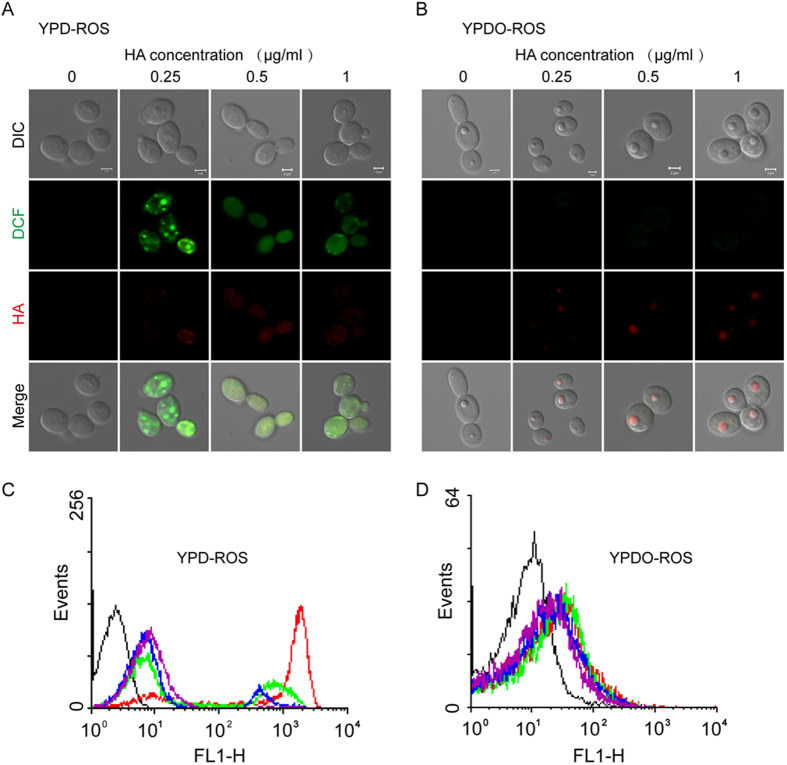
LDs in *C. albicans* cells quench ROS production by trapping assimilated HA. (**A**,**B**) CLSM observation of ROS generation resulting from HA-mediated phototoxins in *C. albicans* cells cultured in YPD (**A**) or YPDO (**B**) medium. HA-treated *C. albicans* cells were stained with DCFHDA, which was rapidly oxidised by ROS into its fluorescent derivative DCF. The green fluorescence intensity of DCF was used to indicate the amount of generated ROS. (**C**,**D**) ROS production for HA-mediated photodynamic inactivation in *C. albicans* cultured in YPD (**C**) or YPDO (**D**) medium, revealed by flow cytometry detection. Black line, control; red line, 0.25 μg/ml of HA; green line, 0.5 μg/ml of HA; blue line, 1 μg/ml of HA; and purple line, 2 μg/ml of HA.

**Figure 5:**
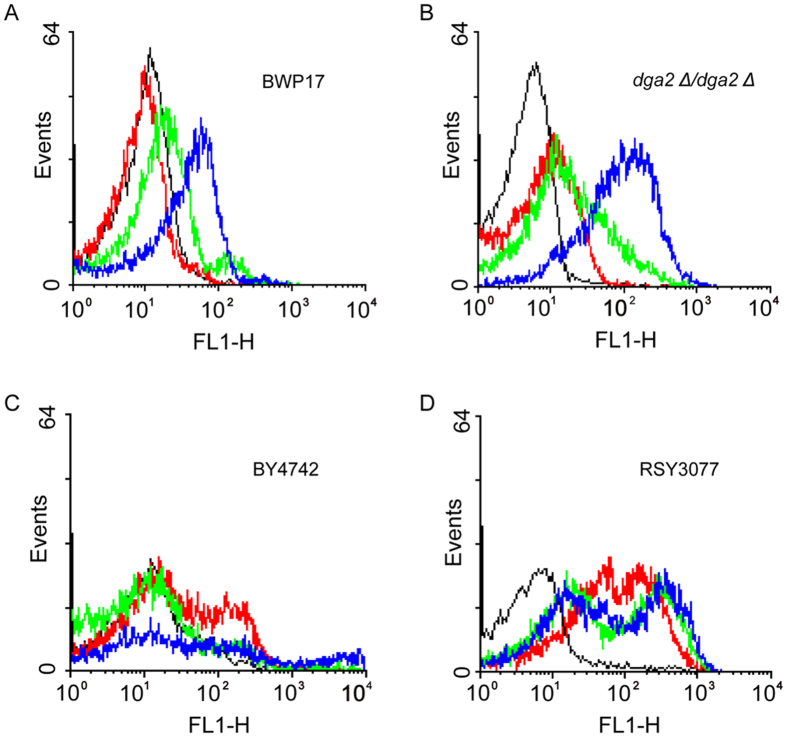
ROS production in yeast mutants defective for LD formation in response to HA-mediated phototoxins. (**A**,**B**) ROS production in *C. albicans* BWP17 (**A**) and *dga2* Δ*/dga2* Δ (**B**) strains that were treated with a series of concentrations of HA and light irradiation, as revealed by flow cytometry detection. Black line, control; red line, 0.25 μg/ml of HA; green line, 0.5 μg/ml of HA; blue line, 1 μg/ml of HA. (**C**,**D**) ROS production in *S. cerevisiae* BY4742 (**C**) and RSY3077 cells with defects in LD formation (**D**) following exposure to a series of concentrations of HA and light irradiation, as detected by flow cytometry. Black line, control; red line, 1 μg/ml of HA; green line, 2 μg/ml of HA; blue line, 4 μg/ml of HA.

Based on the above findings that neutral lipids or lipophilic agents trap HA and quench ROS formation (Figs 1I and 2), we next investigated the effect of HA-mediated phototoxicity on ROS production in *C. albicans* cells. Large amounts of ROS were produced in the HA-treated cells cultured in YPD medium (Fig. 4A,C). However, *C. albicans* cells harboured larger LDs and produced less ROS when cultured in YPDO medium (Fig. 4B,D). The *C. albicans* mutant strain *dga2*Δ*/dga2*Δ, which has a reduced ability to form LDs, generated much more ROS in the presence of HA-mediated photodynamic inactivation compared with its parent strain BWP17, as indicated by DCF fluorescence (Fig. 5A,B). As expected, measurements of HA-mediated phototoxicity in *S. cerevisiae* showed that much lower yields of intracellular ROS were generated in the wild type strain BY4742 compared with the LD-deficient mutant strain (*dga1*Δ *lro1* Δ*are1*Δ *are2*Δ) (Fig. 5C,D). Based on these results, we concluded that LD-induced resistance is a result of the cells escaping oxidative damage by compartmentalising toxins into LDs and the consequent sequestration of ROS formation.

### LD-deficient mutants are more sensitive to some fungicides

Taking into consideration the central roles of LDs in self-resistance to endotoxins and drug resistance to extracellular toxins, we were interested in assessing the impact of yeast LDs on other antibiotics. The susceptibility of *C. albicans* cells cultured in YPD or YPDO medium to clinically-used antifungal agents^36^ and the quorum sensing molecule farnesol^37^,^38^ was tested. We found that the growth inhibition caused by fluconazole (FLC), terbinafine (Ter), caspofungin (CAS), amphotericin B (AmB) and miconazole (Mic) was not significantly different in the cells cultured in the above two culture media (Supplementary Fig. 10A, B). By contrast, *C. albicans* cells with large LDs were resistant to farnesol compared with cells with small LDs, possibly due to differences in intracellular distribution (Supplementary Fig. 10C). By examining the intracellular fluorescence distribution of dansyl-labelled amphotericin B (AmB-Ds)^39^, we found that AmB-Ds was bound to the cytoplasmic membrane and spread throughout the cytoplasm of dead cells rather than localising to specific LDs in *C. albicans* (Supplementary Fig. 11), in accordance with a previous report showing that AmB aggregates in the cell membrane and extracts ergosterol from the lipid bilayers^40^. This suggested that LDs had a smaller effect on the trapping of most of these clinically used antifungal agents. However, AmB or Mic treatment stimulated an increase in the number and size of LDs in *C. albicans* (Supplementary Fig. 12A). A similar result was obtained when farnesol was studied, whereas no significant effects were observed following Ter, CAS or FLC treatments (Supplementary Fig. 12B). These results suggest that the increased size or number of LDs was a stress response to treatment with antifungal agents, although the cells with large LDs did not escape death (Supplementary Fig. 12C). This is consistent with a previous finding that endoplasmic reticulum (ER) stress-inducers, such as tunicamycin and brefeldin A, stimulate LD formation in *S. cerevisiae* but do not guarantee cell survival during ER stress^41^.

Further testing of *dga2*Δ*/dga2*Δ in growth inhibition assays with CAS, FLC, Ter, farnesol, AmB and Mic showed that *dga2*Δ*/dga2*Δ was more susceptible to farnesol, AmB, and Mic than its parent strain BWP17. However, no significant differences were observed between *dga2*Δ*/dga2*Δ and BWP17 in their responses to other drugs (Supplementary Fig. 13). Moreover, susceptibility testing of *S. cerevisiae* showed that the LD mutants were also highly susceptible to farnesol, AmB, and Mic, especially the strains that could not synthesise TAGs (RSY3290) or neutral lipids (RSY3077) (Supplementary Figs. 14 and 15). These data collectively demonstrate that disruption of LD formation renders yeast cells more susceptible to some antifungal agents, although LD-induced resistance was not a universal response to all types of drugs.

## Discussion

Our findings reveal that LDs can trap endogenous or external lipophilic phototoxins and this is a resistance mechanism that protects against toxins in both the producer and the recipient. Starting with an investigation of self-resistance mechanisms in an endolichenic fungus, *Phaeosphaeria* sp., that biosynthesizes phototoxic PQs, we found that the toxins were trapped inside LDs in the producer cells (Fig. 1G). The toxicity of PQs results from ROS formation, particularly singlet oxygen production, following exposure to light. When HA, a PQ derivative, was dissolved in lipophilic solvents that contained triglycerides, fewer singlet oxygen molecules were generated by light irradiation (Fig. 1I), suggesting that trapping PQs into LDs conferred resistance to endogenous phototoxins. This LD-induced resistance mechanism is also critical for maintaining a lichen community that contains a toxin producer.

Moreover, yeast cells with larger LDs trap HA and display higher resistance to phototoxicity, suggesting that trapping toxins in LDs is also a mechanism for the recipient to resist extracellular toxins. The reduced phototoxic effect of HA in *C. albicans* and *S. cerevisiae* can be attributed to the ability of LDs to trap toxins, and yeast with larger LDs may trap more toxins, thus conferring increased resistance to HA-mediated phototoxicity or to the lipophilic agent farnesol. The trapped toxins in LDs lose the ability to induce the production of ROS, resulting in the increased survival of the fungi (Figs 2E and 4). This is different from what occurs in organelles such as chloroplasts, mitochondria and peroxisomes that employ antioxidant defence systems to protect against ROS^8^. Our findings demonstrate that LDs could be a novel strategy for relieving oxidative stress via trapping of toxins. This observation is supported by the fact that yeast mutants with depleted LD formation are more susceptible to oxidative stress resulting from photodynamic inactivation (Fig. 3).

In nature, organisms have evolved multiple strategies for adapting to environmental challenges and, in particular, for counteracting oxidative stress^42^,^43^. Cells possess very efficient enzymatic and non-enzymatic antioxidant defence systems that work together to protect themselves from oxidative damage^8^,^9^. The non-enzymatic antioxidant defence systems mainly comprise physical quenching and chemical quenching processes that sequester ROS production^44^,^45^,^46^. Physical quenching is the main mode for deactivating lipid-soluble antioxidants and is related to energy transfer^44^, while chemical quenching prevails in water-soluble antioxidants, and the quencher molecule is generally oxidised or consumed in the process of ROS scavenging^44^. The present findings contribute to our understanding of how LD-mediated trapping leads to the inactivation of the ROS production that generally occurs in response to lipophilic toxins. The main constituents of LDs include TAGs and SEs, which have lipophilic characteristics that give them high affinity to PQs and lay the basis for trapping mechanisms. For large amounts of toxins, trapping in LDs is probably the best resistance mechanism for preventing self-damage.

Unexpectedly, *C. albicans* cells with large LDs displayed no obvious alterations in susceptibility in response to most of the tested antibiotics compared with those with small LDs following culture in YPD medium (Supplementary Fig. 10). This was probably owing to the polarity of tested antibiotics, which prevents them from dissolving mutually with LDs or to the specificity of their targets outside LDs, as exemplified by the fact that AmB-Ds primarily bind to the cytoplasmic membrane rather than being taken up into LDs (Supplementary Fig. 11). However, the increases in LD size and number observed in *C. albicans* cells that were stimulated by AmB or Mic treatment suggested that the generation of neutral lipids is a stress response. Disruption of *DAG2* caused the loss of the ability to form LDs and rendered *C. albicans* more susceptible to AmB, Mic and farnesol treatment (Supplementary Fig. 13). Moreover, the LD-defective strain of *S. cerevisiae* was highly susceptible to farnesol, AmB and Mic (Supplementary Fig. 14 and Fig. 15). This implies that the disruption of LD formation results in a suppressed stress response and renders yeast cells more susceptible to fungicides.

In summary, our investigation of the self-protecting strategies of an endolichenic fungus that involve the trapping of phototoxins led to the determination that fungal LDs play crucial roles in drug resistance and adaptations to stress. Although it remains to be determined whether this represents a more generalised strategy for resistance or self-protection in other organisms, our findings offer new insights into the roles of LDs in resistance mechanisms used by fungi and the development of antifungal strategies.

## Methods

### Lichen studies

Lichen (*Heterodermia obscurata* (Nyl.) trevis) was collected from Yunnan province, People’s Republic of China. The lichen thallus was sterilised and cut transversely or longitudinally into sections for CLSM observation. Longitudinal z-stack observations were acquired using 30 confocal sections that covered the entire depth of the lichen. PQs produced by the endolichenic fungus identified as *Phaeosphaeria* sp^25^. were excited by 488 nm lasers and recorded in an emission spectrum of 400–603 nm for yellow fluorescence and 603–700 nm for red fluorescence. Fixed maximum-intensity projections and stacks of images were generated with Zeiss 2010 software. *Phaeosphaeria* sp. was cultured on potato dextrose agar (PDA) plates in dark or under light irradiation. Each day, the growth of the colonies was recorded by imaging. After 7 days, a sample of the organisms was scraped from the colonies under light irradiation and suspended in PBS solution for CLSM observation with a Zeiss LSM700 confocal microscope. The samples were also stained with BODIPY at a final concentration of 1 μg/ml for confocal microscopic observations. BODIPY and PQs were excited by 488 nm and 555 nm lasers, respectively, and recorded in an emission spectrum of 490–550 nm for green fluorescence and 570–700 nm for red fluorescence.

### Measurement of singlet oxygen production generated by HA in different solvents

The amount of ^1^O_2_ was measured using 9,10-diphenylanthracene, which specifically reacts with ^1^O_2_ to form an endoperoxide accompanied by a decrease in absorbance at 355 nm, indicating the production of ^1^O_2_^47^. Briefly, HA was dissolved in petroleum ether, liquid paraffin and n-heptane (3:1), n-heptane, dichloromethane, chloroform or acetone, at a final concentration of 4 μg/ml and separated into two groups. Triglyceride mix (Sigma-Aldrich, catalogue No. 17811-1AMP) was added to the chloroform or acetone at a final concentration of 10 mg/ml. The addition of 20 μg/ml 9,10-diphenylanthracene was used to trap the ^1^O_2_ produced by HA. One group was irradiated by light for 30 min, and the other group was maintained in darkness. Then, the absorbance of each sample was measured at 355 nm using an ultraviolet–visible spectrophotometer (UV-2550, Shimadzu Japan). The decreased percentage was calculated to indicate the production of ^1^O_2_ generated by HA.

### Measurement of octanol-water partition coefficients

The octanol-water partition coefficients of HA were determined according to a previously described method^48^. Briefly, HA was dissolved in a mutually saturated octanol and water mixed solution. The concentration of HA in octanol and water was detected by high-performance liquid chromatography (HPLC) using an Agilent 1260 system. The HA was separated on an Eclipse XDB-C18 column (5 μm, 150 mm × 2.1 mm I.D., Agela. Science Inc., USA) through a 4 mm × 3 mm pre-column (Security Guard C18 cartridge, Phenomenex, Inc.) that was maintained at room temperature. The mobile phase consisted of acetonitrile/water (80:20, v/v) and was set at a flow rate of 0.80 ml/min. The concentration of HA dissolved in octanol or water was calculated by comparison with the standard curve.

### Yeast strains and growth conditions

The *Saccharomyces cerevisiae* wild-type strain BY4742 and the LD-defective strains RSY3290, RSY1826, and RSY3077 were cultured in YPD medium (1% yeast extract, 2% bacto peptone and 2% dextrose) at 30 °C with rotational shaking at 200 rpm. *C. albicans* wild type strains were cultured in YPD, YPDO medium (1% yeast extract, 2% bacto peptone, 2% dextrose and 0.1% sodium oleate (Sigma-Aldrich)) or SD medium (0.67% nitrogen base and 2% dextrose with all amino acids supplemented). *C. albicans* mutant strains were grown in YPD medium supplemented with the necessary auxotrophic requirements, including 80 mg/L uridine, 20 mg/L histidine, and 40 mg/L arginine. The genotypes of the yeast strains used are listed in Supplementary Material Tables S1 and S2.

### Strain construction

The mutant strains deficient in LDs formation were generated in BWP17 using PCR deletion cassettes, as described previously^49^. The primers used are provided in Supplementary Material Table S3.

### Photodynamic inactivation by HA in *C. albicans* or *S. cerevisiae*

*C. albicans* or *S. cerevisiae* strains were cultured in YPD medium at 30 °C overnight. Then, 1 ml aliquots of yeast suspensions (2 × 10^6^ CFUs/ml) in YPD medium were incubated in glass tubes with HA at final concentrations ranging from 0.125 to 4 μg/ml. The samples were placed under a 9 W fluorescent lamp with an irradiation intensity of 10 W/m^2^, which was detected using a photometer. Aliquots of 100 μl were removed at the indicated times and spread on YPD plates to determine the surviving CFUs.

### Colocalisation studies

*C. albicans* SC5314 was grown in SD medium overnight. Cells were collected and stained with BODIPY 493/503 (5 μM) or CMAC (5 μM) for 30 minutes at room temperature (RT) to determine the localisation of LDs and vacuoles. Prestained cells were washed and incubated with HA (2 μg/ml) in the dark at room temperature (RT) for 10 min. To stain the nucleus, cells were pre-incubated with HA (2 μg/ml) in the dark at RT for 10 min and then fixed with 70% ethanol. Fixed cells were stained with 2.5 μg/ml of 4′, 6-diamidino-2-phenylindole (DAPI) to indicate the location of nuclei. Stained cells were washed and then observed by CLSM. Argon lasers at 405 nm, 488 nm and 555 nm were used to excite CMAC/DAPI, BODIPY, and HA, respectively.

To observe the distribution of amphotericin B in *C. albicans* cells, dansyl-labelled amphotericin B (AmB-Ds) was synthesised according to a previously reported method^39^. *C. albicans* SC5314 cells were treated with 30 μg/ml of AmB-Ds for 12 h. Cells were then collected and washed following with PI (10 μg/ml) staining for CLSM observation. AmB-Ds and PI were excited by 405 nm and 555 nm, respectively, and recorded in an emission spectrum of 430–530 nm for blue fluorescence and 590–700 nm for red fluorescence.

### The time-lapse observation of yeast death

*C. albicans* SC5314 was cultured overnight in YPD or YPDO medium. The collected cells were incubated with HA (2 μg/ml) for 2 min in YPD medium containing SYTOX at a final concentration of 5 μM to observe cell death. Then, cells were exposed to light irradiation with a maximum intensity under a Zeiss Confocal Laser Scanning Microscope LSM700. We manually imaged cell states at 1 or 2 min intervals.

### Susceptibility testing of yeast to antifungal agents

*C. albicans* SC5314 cells were cultured in YPD medium or YPDO medium at 30 °C overnight. Cells were then collected and adjusted to 2 × 10^5^ CFUs/ml with YPD or YPDO medium. *C. albicans* mutant strains and LD formation-defective *S. cerevisiae* strains were cultured in YPD medium. They were tested for their susceptibility to antifungal agents including fluconazole (FLC), caspofungin (CAS), terbinafine (Ter), miconazole (Mic), amphotericin B (AmB) and farnesol. After 6 or 12 h of treatment, the cells were serially diluted and spotted onto YPD agar plates. After 48 h of incubation at 30 °C, the plates were imaged, and the inhibitory effect was determined by colony counting.

### Quantitative real-time PCR

*C. albicans* SC5314 was cultured in YPD or YPDO medium overnight for RNA extractions. Measurement of the relative quantitative expression of target genes was conducted using an Eppendorf Mastercycler Real-time PCR System^50^. Relative gene expression was calculated using the formula 2^−ΔΔCT^. The primers used for *ARE2*, *DGA2*, *LRO1* and *ACT1* in this test are listed in Supplementary Material Table S3. *ACT1* was used as an internal reference gene.

### Measurement of LD size

Cells were stained with BODIPY (5 μM) for 30 min or with Nile Red (10 μg/ml; Sigma-Aldrich, St Louis, MO) for 5 minute, two probes of indicating LDs, at room temperature and washed twice with PBS for confocal microscopic observation. The cells were also subjected to a FACScan flow cytometer (Becton Dickinson, San Jose, CA) with excitation at 488 nm to measure fluorescence intensity, which was indicative of LD size. The resultant data were processed with WinMDI 2.9 software.

### Intracellular ROS measurement

*C. albicans* SC5314 or *S. cerevisiae* BY4742 was cultured overnight in YPD or YPDO medium. For LD mutants, cells were cultured in YPD overnight. Then, cells were collected and diluted to 10^6^ cells/ml and incubated with HA in YPD medium for 2 min. The *C. albicans* cells were exposed to 10 min of light irradiation, and the *S. cerevisiae* cells were exposed to 20 min of light irradiation. Then, cells were collected by centrifugation and stained with 40 μg/ml DCFHDA. The loaded DCFHDA was converted into the membrane-impermeable agent DCFH by intracellular esterase. DCFH is rapidly oxidised by ROS into its fluorescent derivative, DCF. The green fluorescence intensity of DCF was used to indicate the amount of ROS production. The stained samples were subject to flow cytometry detection or confocal microscopic observation with excitation at 488 nm and emission in the range of 510–560 nm to measure the ROS levels.

### Statistical Analysis

One-way ANOVA with the Bonferroni test was used to compare the survival time of *C. albicans* cells cultured in YPD or YPDO medium. Other data were analysed by Student’s *t* test. Statistical results are presented as the means ± SD. Asterisks indicate critical levels of significance (*p < 0.05, **p < 0.01, and ***p < 0.001).

## Additional Information

**How to cite this article**: Chang, W. *et al.* Trapping toxins within lipid droplets is a resistance mechanism in fungi. *Sci. Rep.* **5**, 15133; doi: 10.1038/srep15133 (2015).

## Supplementary Material

Supplementary Information

Supplementary Video 1

Supplementary Video 2

Supplementary Video 3
