# Supplementary material for: Trapping toxins within lipid droplets is a resistance mechanism in fungi
Source: Sci Rep. 2015 Oct 14;5:15133. doi: 10.1038/srep15133 (PMC4604559; doi:10.1038/srep15133)
Supplement: Supplementary Information [file srep15133-s1.pdf]

Supplementary Information

Trapping toxins within lipid droplets is a resistance mechanism in fungi

Wenqiang Chang,[1] Ming Zhang,[1] Sha Zheng,[1] Ying Li,[1] Xiaobin Li,[1] Wei Li,[1] Gang Li,[1] Zhaomin Lin,[1] Zhiyu Xie,[1] Zuntian Zhao[2] and Hongxiang Lou[1]*

[1] Department of Natural Product Chemistry, Key Lab of Chemical Biology of Ministry of Education, Shandong University, No. 44 West Wenhua Road, Jinan City, Shandong Province, China

[2] College of Life Sciences, Shandong Normal University, No. 88 East Wenhua Road, Jinan City, Shandong Province, China

*Corresponding author: Hongxiang Lou; E-mail: louhongxiang@sdu.edu.cn

Supplemental Experimental Procedures

Strain Construction

For construction of GFP labeled strains, the fragments were PCR amplified from pGFP–URA3 using primer pairs of LRO1-GFP-F/ LRO1-GFP-R, ARE2-GFP-F/ ARE2-GFP-R, or DGA2-GFP-F/ DGA2-GFP-R containing sequence homologous to *LRO1*, *ARE2*, and *DGA2*, respectively. The PCR fragments were transformed into CAI4. The Ura+ positive transformants were PCR tested for proper integration of the genomes using primer pairs of LRO1-GFP-CH-F/ ARE2-GFP-CH-F/ DGA2-GFP-CH-F and GFP-CH-R. However, the generated GFP labeled strains including LRO1-GFP-CAI4, ARE2-GFP-CAI4 and DGA2-GFP-CAI4 produced faint fluorescence intensity under the fluorescence microscopic observation.

For construction of *dga2* mutant strains, the fragments were PCR amplified from pGEM-URA3 or pGEM-HIS1 using primer pairs of DGA2-D-F/ DGA2-D-R containing sequence homologous to *DGA2*. The PCR fragment derived from pGEM-URA3 was transformed into BWP17. The Ura+ positive transformants were PCR tested for proper integration of the genomes using primer pairs of DGA2-CH-F and URA3-CH-R. The heterozygote of *dga2* mutant strain was obtained. The PCR fragment derived from pGEM-HIS1 was transformed into *dga2* heterozygote for the second round of gene deletion. The His+ positive transformants were confirmed by diagnostic PCR for proper integration of the genomes using primer pairs of DGA2-CH-F and HIS1-CH-R. The *lro1* and *are2* homozygote mutant strains were achieved using the same method.

Supplementary Results

Supplementary Table 1 *C. albicans* strains used in this study.

Strain name	Description	Genotype	Source
SC5314	wild type strain	Prototroph	Gillum *et al.*,1984 [1]
CA2	clinical derived azole sensitive strain		Sun *et al.*,2009 [2]
CA10	clinical derived azole resistant strain		Sun et al.,2009 [2]
BWP17	Ura-, His-,Arg-	*ura3Δ::λimm434/ura3Δ::λimm434 his1::hisG/his1::hisG arg4::hisG/arg4::hisG*	Wilson *et al.*,1999 [3]
lro1Δ/lro1Δ	lro1 homozygote	*ura3Δ::λimm434/lro1Δ::URA3 his1::hisG/lro1Δ::HIS1 arg4::hisG/arg4::hisG*	This study
are2Δ/are2Δ	are2 homozygote	*ura3Δ::λimm434/are2Δ::URA3 his1::hisG/are2Δ::HIS1 arg4::hisG/arg4::hisG*	This study
dga2Δ/dga2Δ	dga2 homozygote	*ura3Δ::λimm434/dga2Δ::URA3 his1::hisG/dga2Δ::HIS1 arg4::hisG/arg4::hisG*	This study
CAI4	Ura-	*ura3Δ::λimm434/ura3Δ::λimm434*	This study
LRO1-GFP-CAI4	*LRO1* labeled with GFP in CAI4	*ura3Δ::λimm434/LRO1-GFP-URA3*	This study
ARE2-GFP-CAI4	*ARE2* labeled with GFP in CAI4	*ura3Δ::λimm434/ARE2-GFP-URA3*	This study
DGA2-GFP-CAI4	*DGA2* labeled with GFP in CAI4	*ura3Δ::λimm434/DGA2-GFP-URA3*	This study

Supplementary Table 2 ***S. cerevisiae*** **strains used in this study.**

Strain name	Description	Genotype	Source
BY4742	wild type strain	MATα; *his3*Δ1; *leu2*Δ0; *lys2*Δ0; *ura3*Δ0	Euroscarf
RSY3290	*lro1Δ dga1Δ*	MATα; *his3*Δ1; *leu2*Δ0; *lys2*Δ0; *ura3*Δ0; *lro1*::KanMX; *dga1*::lox-His-lox	Vineet Choudhary [4]
RSY1826	*are1Δ are2Δ*	MATα; *his3*Δ1; *leu2*Δ0; *lys2*Δ0; *ura3*Δ0; *are1*::His5; *are2*::kanMX	René Köffel [4]
RSY3077	*lro1Δ dga1Δ are1Δ are2Δ*	MATα; *his3*Δ1; *leu2*Δ0; *lys2*Δ0; *ura3*Δ0; *met15* Δ0; *are1*::kanMX*; are2*::kanMX*; trp*::URA*; lro1*::TRP; *dga1*::lox-His-lox	Vineet Choudhary [4]

Supplementary Table 3 Primers used in this study.

Primers	Sequences (5'-3') a
LRO1-F	TGATTTCTACTGGGTTAGAA
LRO1-R	GTAGTGGTATCCAACATGAT
ARE2-F	TGCTTGGATTTCCCATGGATT
ARE2-R	TTGGTGTGTCCTTTTGGTAATG
DGA2-F	TAAACATGCCAGAGTTGGTT
DGA2-R	TTAACTTCACGCTTAGATGG
ACT1-F	TCCAGAAGCTTTGTTCAGAC
ACT1-R	TGCATACGTTCAGCAATACC
LRO1-GFP-F	**CACACGATACAGAATCGATACGTTAGCAATTTGAAGAAAATTGTAGAAAATATGCATTTG**GGTGGTGGTTCTAAAGGTGAAGAATTATT
LRO1-GFP-R	**ACCAATAATTATCAATACCCAATTATTTCTGGAAAACAGTATGGTAGTTTAAGTGACTCT**TCTAGAAGGACCACCTTTGATTG
LRO1-GFP-CH-F	TGTAATTGATGCTGATTCTCC
ARE2-GFP-F	**ATTTGTTGGTTTGGTTTTATTAGCGGTCCAAGTATTATTTGTACACTTTATTTAGTGTTT**GGTGGTGGTTCTAAAGGTGAAGAATTATT
ARE2-GFP-R	**ATACAATATGTATTGATTGGAACGCCATTCTTATGACACTTTAATTATTACACTTCCATA**TCTAGAAGGACCACCTTTGATTG
ARE2-GFP-CH-F	TGATTTGGGATGCCATTCTT
DGA2-GFP-F	**GTCTATGAAGAGAATAAACATAAGTTTGGTTATGGAGATGTCGAATTCAGTATCGTCGAA**GGTGGTGGTTCTAAAGGTGAAGAATTATT
DGA2-GFP-R	**AAATTAGTTATAGAGGTCATAAAATTGTGATTTTGATTCTCAAAAGCTGTGTTTGATGAC**TCTAGAAGGACCACCTTTGATTG
DGA2-GFP-CH-F	TAAACATGCCAGAGTTGGTT
GFP-CH-R	CAATTTACCGTAAGTAGCATCAC
ARE2-D-F	**AGAACAAATACACTGGACCAGTTGAATGCTATCTCCGATAAGAATACCAAAAGGAAATCT**GTTTTCCCAGTCACGACGTT
ARE2-D-R	**ATTTGGAATAATAACAAATAGCCTCTCAAAGTTCCAAAAATGACATACATCACAAGCTCG**TGTGGAATTGTGAGCGGATA
ARE2-CH-F	CATATACGTATACGCATCGTA
DGA2-D-F	**TTGCTTGCAATGACAGACACATCTGACCTTAAACCAGAACATACAGAGAAGGCTACAGGG**GTTTTCCCAGTCACGACGTT
DGA2-D-R	**TACTGAATTCGACATCTCCATAACCAAACTTATGTTTATTCTCTTCATAGACATTTCTCA**TGTGGAATTGTGAGCGGATA
DGA2-CH-F	CGTCTTATAATCTTGATTGAGTG
LRO1-D-F	**ACTTCAAACATGTCAGGTTTGAAAAATAGAAAATCCACTATTAATAAGGACCAAAATGCA**GTTTTCCCAGTCACGACGTT

LRO1-D-R	**CTAAACATTAATTACTCTTCCTTTCTTCCTTTCCTCTCCTTTTGGTCTATTACAAATGCA**TGTGGAATTGTGAGCGGATA
LRO1-CH-F	CAACACAATCAAGACAAACTTT
URA3-CH-R	GCATGAGTTTCTGCTCTCTCAC
HIS1-CH-R	CTTCTAAACTGTATATCGGCA

a: The bold font indicates the homologous sequences with target genes.

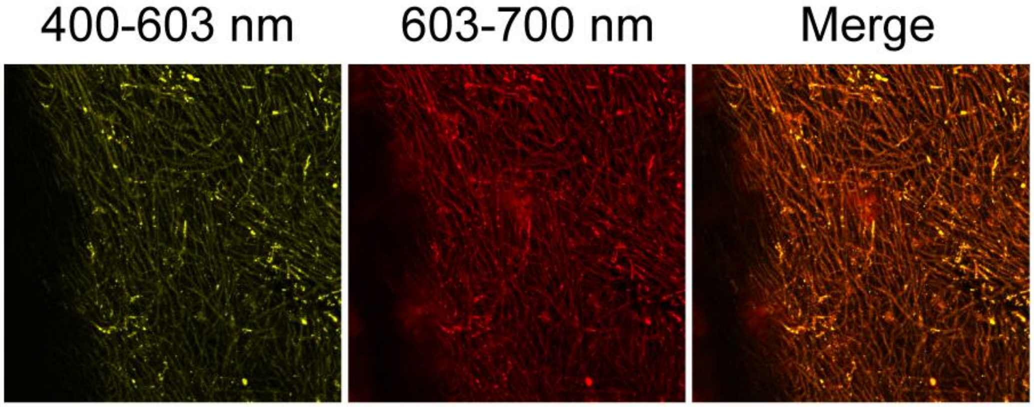

Supplementary Figure 1. The projection of lichen *Heterodermia obscurata* (Nyl.) trevis thallus that was longitudinally cut and observed by CLSM using the z-stack mode. The lichen thallus was longitudinally cut and z-stack observations were acquired using 30 confocal sections that covered the entire depth of the lichen. The scanning images of 30 sections are provided in Supplementary Video 1. The stacks of images were fixed maximum intensity projection as a still image. The fluorescent agents within the filaments were excited by 488 nm lasers and recorded in an emission spectrum of 400-603 nm for yellow fluorescence and 603-700 nm for red fluorescence.

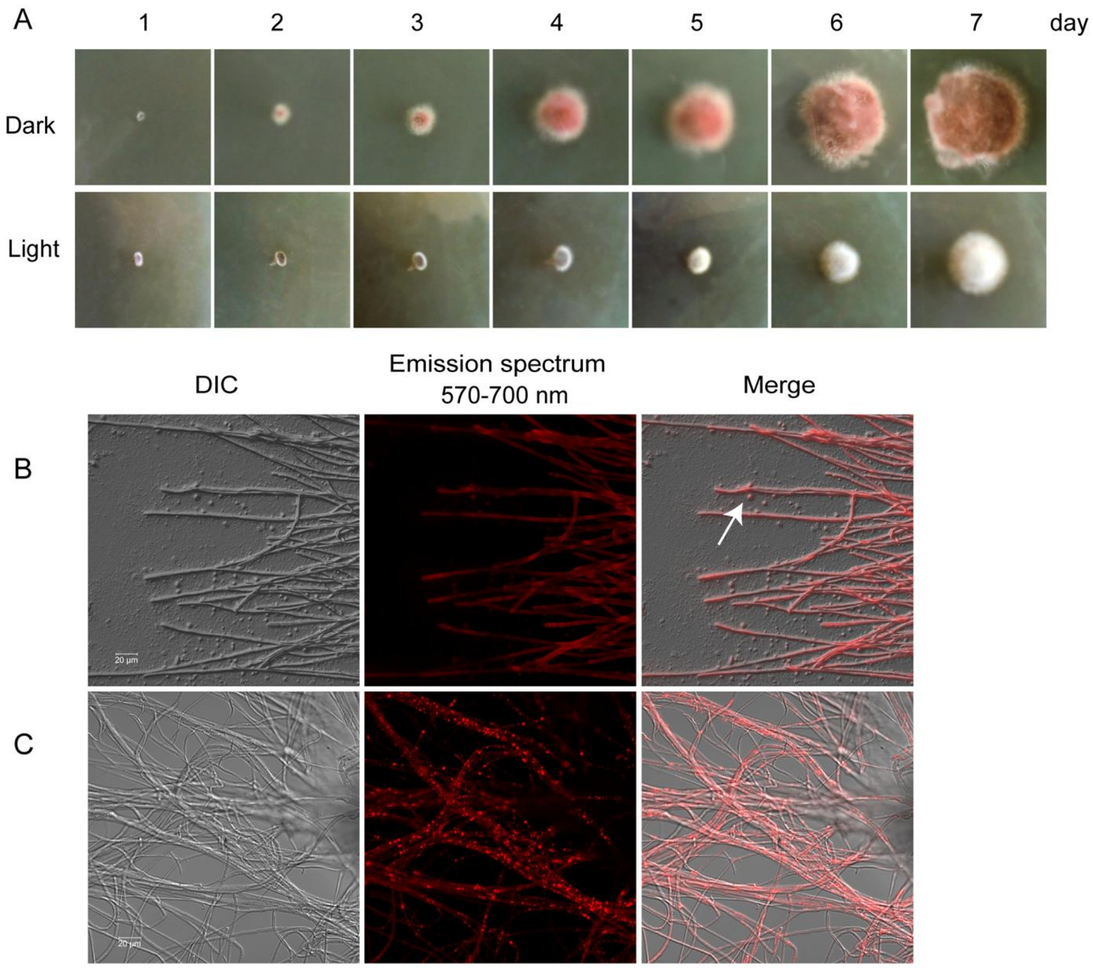

Supplementary Figure 2. Analysis of the growth state of endolichenic fungus *Phaeosphaeria* sp. and the distribution of its generated pigments in two culture conditions. (**A**) Comparation of the growth state of *Phaeosphaeria* sp. cultured in dark or under light irradiation on PDA plates. (**B**) The fungal filaments were full of pigments with red fluorescence when *Phaeosphaeria* sp. was cultured on PDA plate in the darkness as revealed by CLSM observation. The pigment granules with red fluorescence were secreted by the fungus. One secreted pigment granule was indicated by the white arrow. (**C**) The pigments with red fluorescence accumulated and formed particles within the filaments when *Phaeosphaeria* sp. was exposed to light.

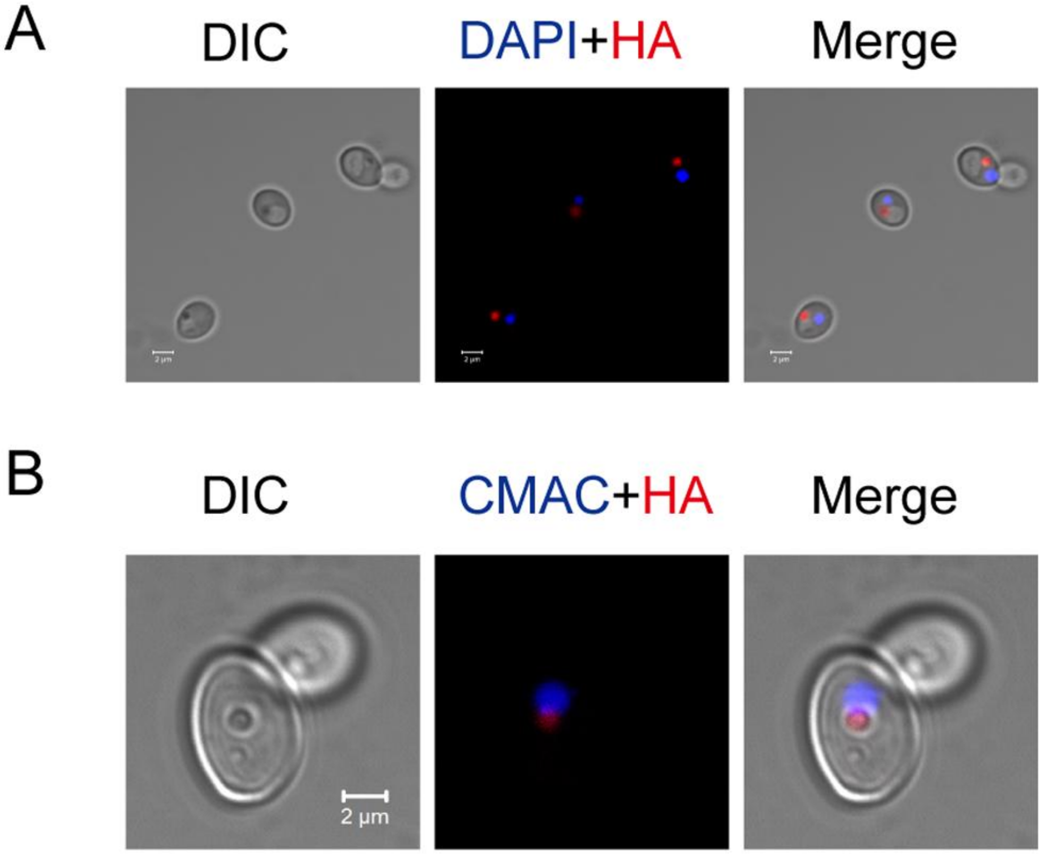

Supplementary Figure 3. The colocalisation of intracellular distribution of HA with the nucleus or vacuoles, as indicated by the staining of DAPI (A) or CMAC (B).

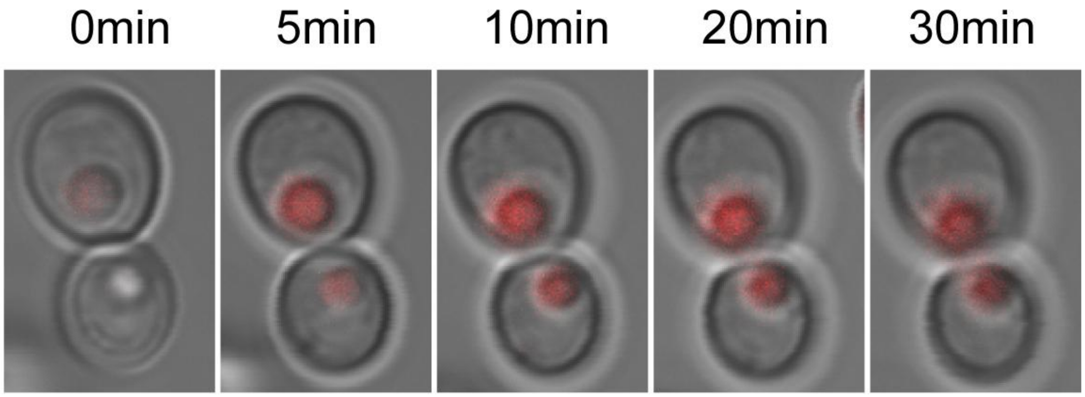

Supplementary Figure 4. The time-sequence observation of HA, which was trapped by LDs in *C. albicans*. *C. albicans* SC5314 was cultured in SD medium and incubated with 2 μg/ml of HA in the darkness. At indicated time, the image was taken by CLSM.

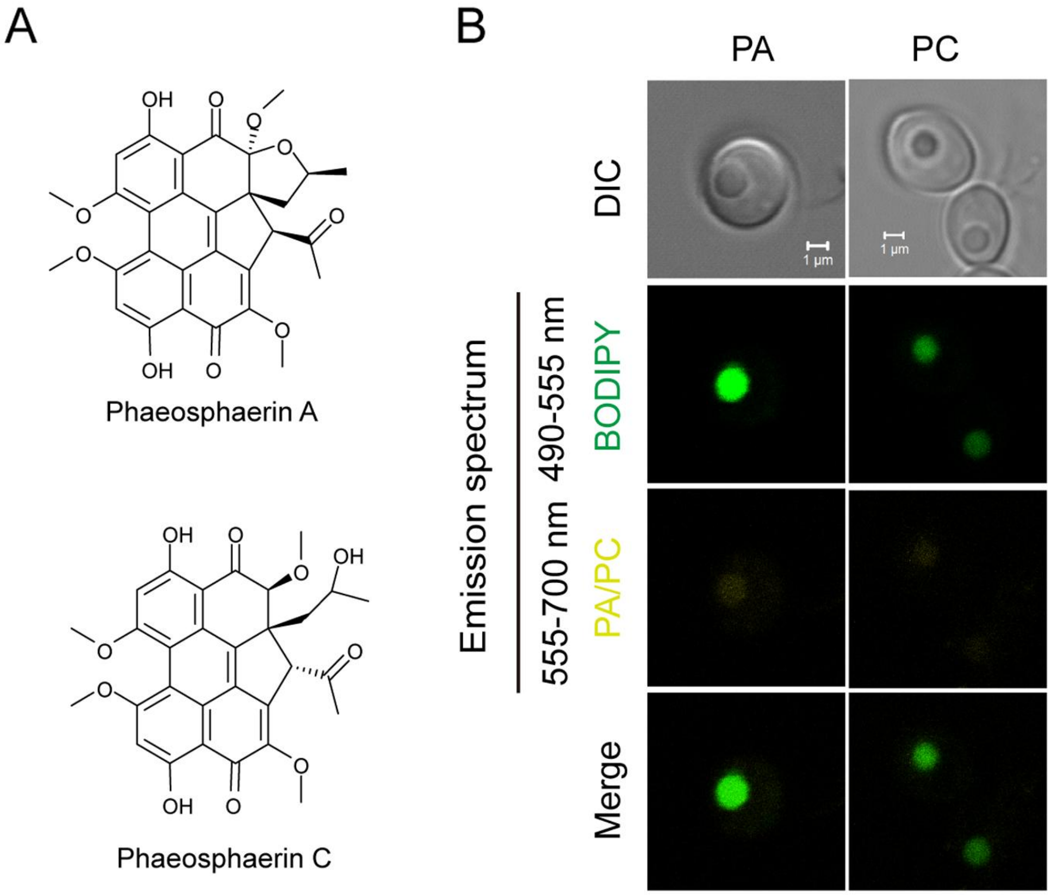

Supplementary Figure 5. The intracellular distribution of PQ derivatives in *C. albicans*. (**A**) The chemical structures of two PQ derivatives phaeosphaerin A (PA) and phaeosphaerin C (PC). (**B**) The colocalisation of PA/PC and LDs. *C. albicans* SC5314 cells were cultured in SD medium and stained with BODIPY, a dye specific for LDs staining. Prestained cells were incubated with PA (8 μg/ml) or PC (8 μg/ml) for 10 min in the darkness and subject to CLSM observation.

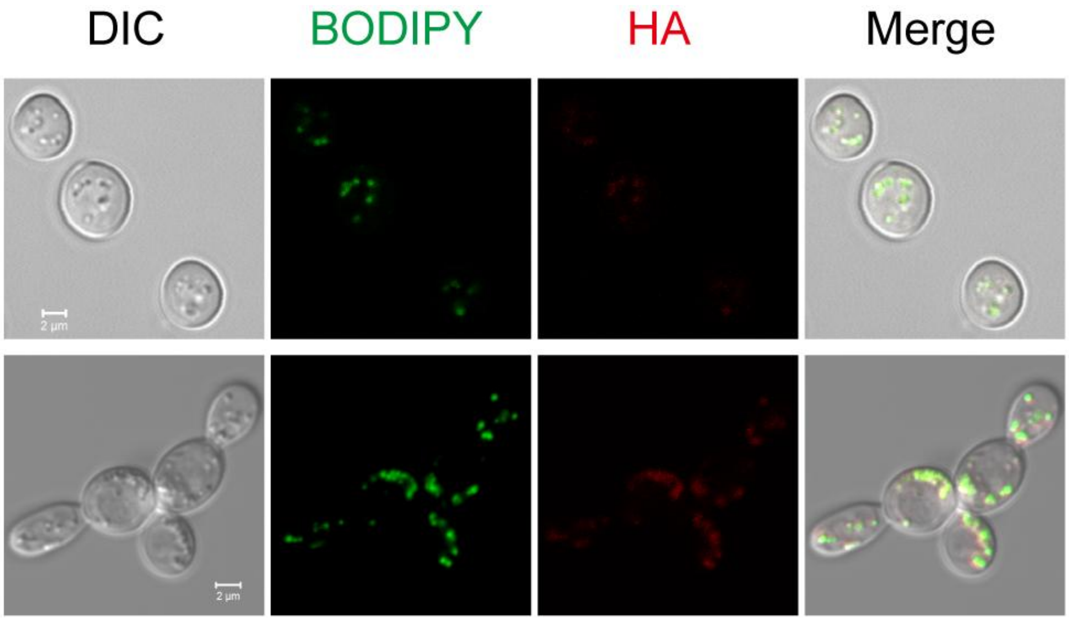

Supplementary Figure 6. The intracellular distribution of HA in *S. cerevisiae*. *S. cerevisiae* BY4742 cells were cultured in YPD medium and stained with BODIPY to indicate the localisation of LDs. Prestained cells were incubated with HA for 10 min in the darkness and subject to CLSM observation.

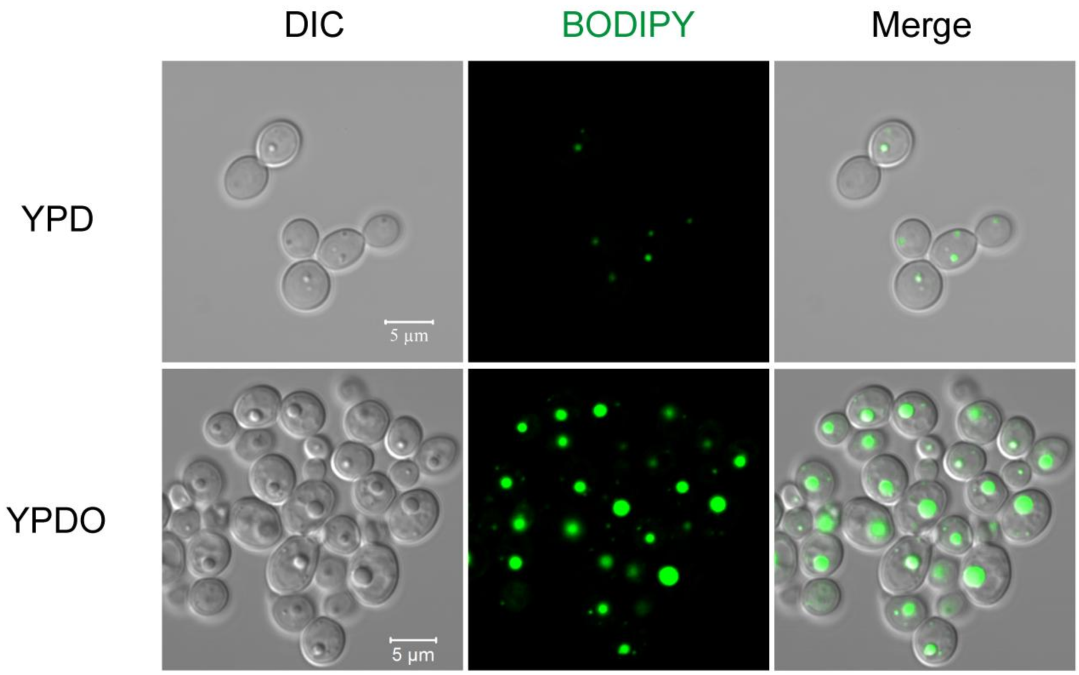

Supplementary Figure 7. The LD size of *C. albicans* increased by the oleate addition as revealed by CLSM observation. *C. albicans* SC5314 was cultured in YPD or YPDO (YPD medium containing oleate) medium overnight. Cells were stained with BODIPY for 30 min and observed by CLSM.

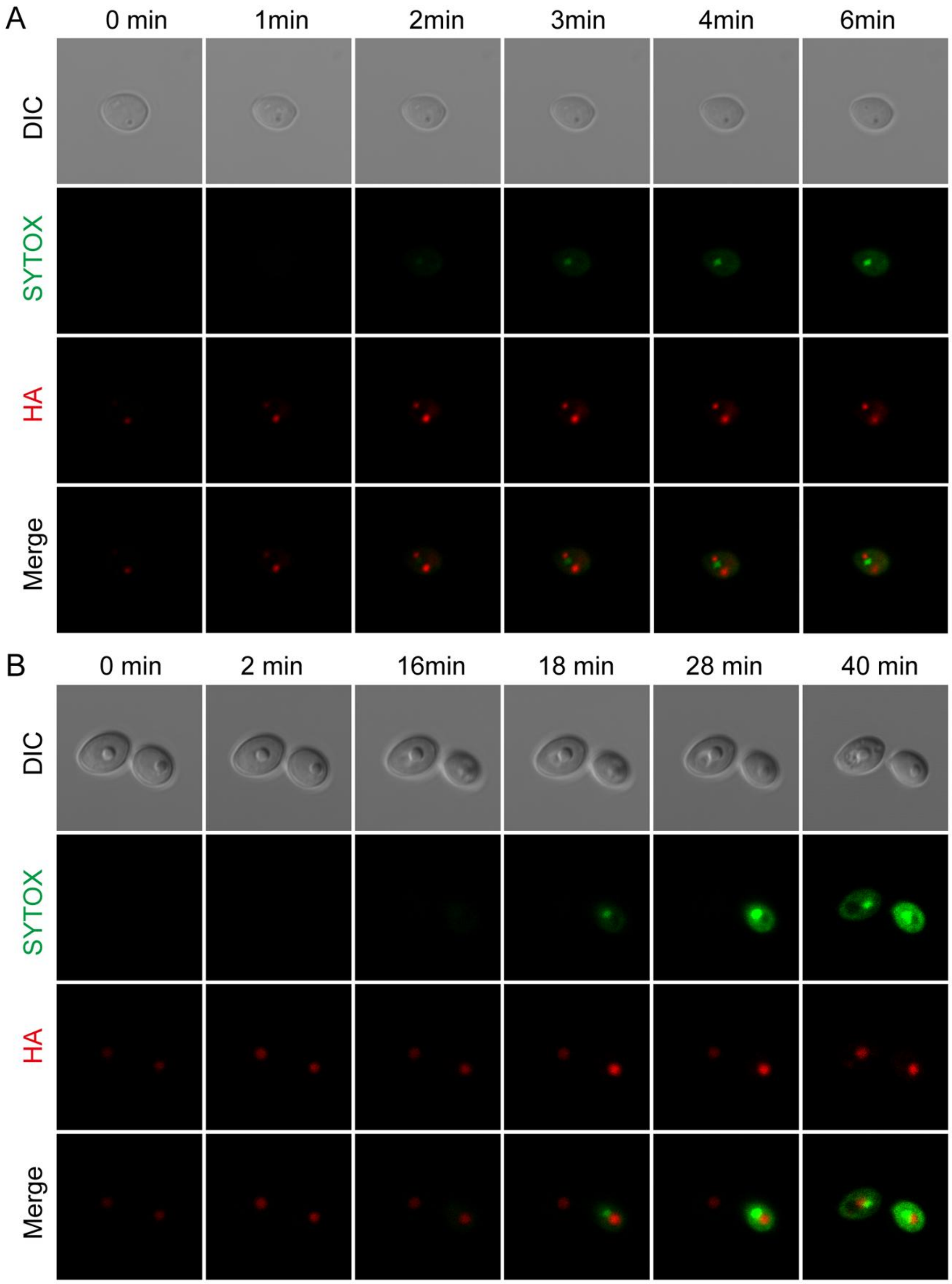

Supplementary Figure 8. **Time-lapse monitoring cell fate of *C. albicans* when cells were treated with HA and exposed to light irradiation.** *C. albicans* SC5314 was cultured in YPD (**A**) or YPDO (**B**) medium and stained with SYTOX, which indicates the live/dead cell state. Cells were exposed to 2 μg/ml of HA for light irradiation and observed by a Zeiss Confocal Laser Scanning Microscope LSM700.

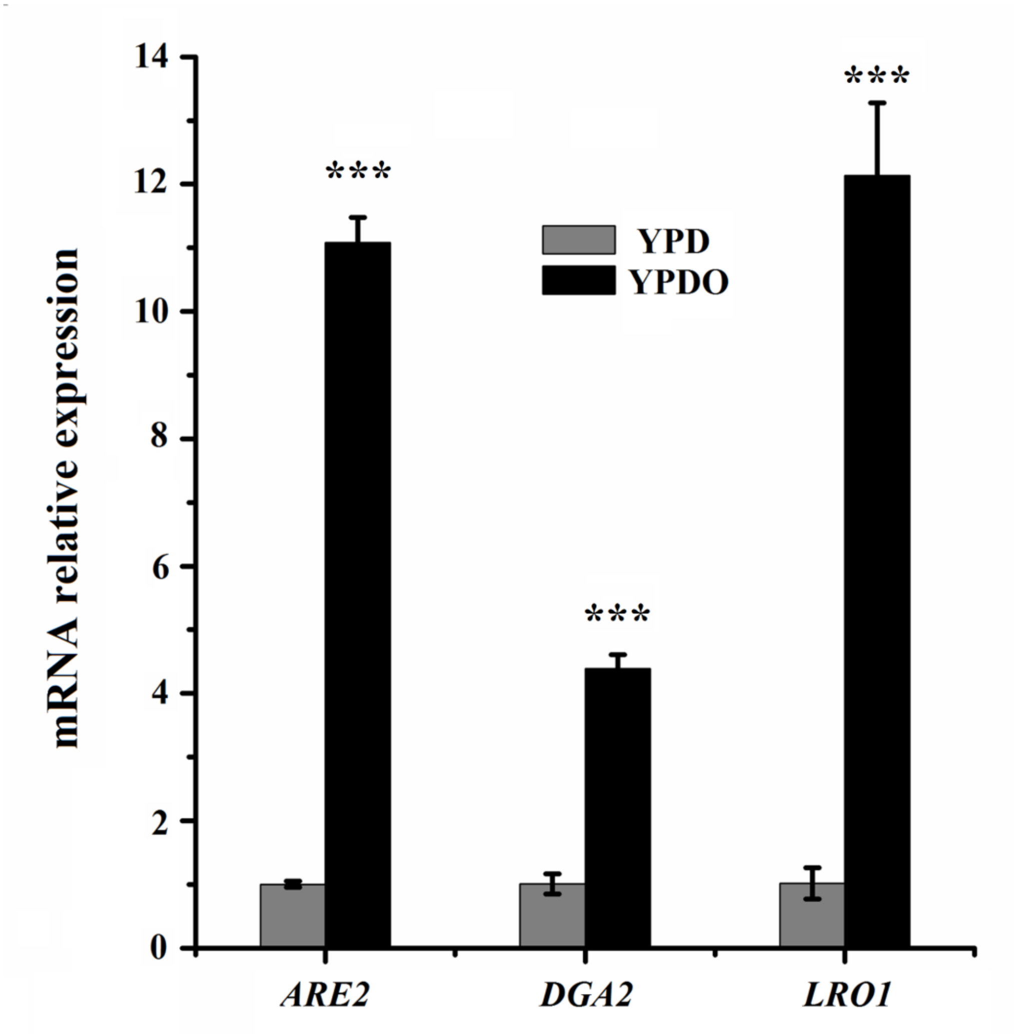

Supplementary Figure 9. Measurement of the relative quantitative expression of LD formation related genes by quantitative real-time PCR. *C. albicans* SC5314 was cultured in YPD or YPDO medium. Cells were collected for RNA extraction. The relative mRNA levels of indicated genes were detected using quantitative real-time PCR.

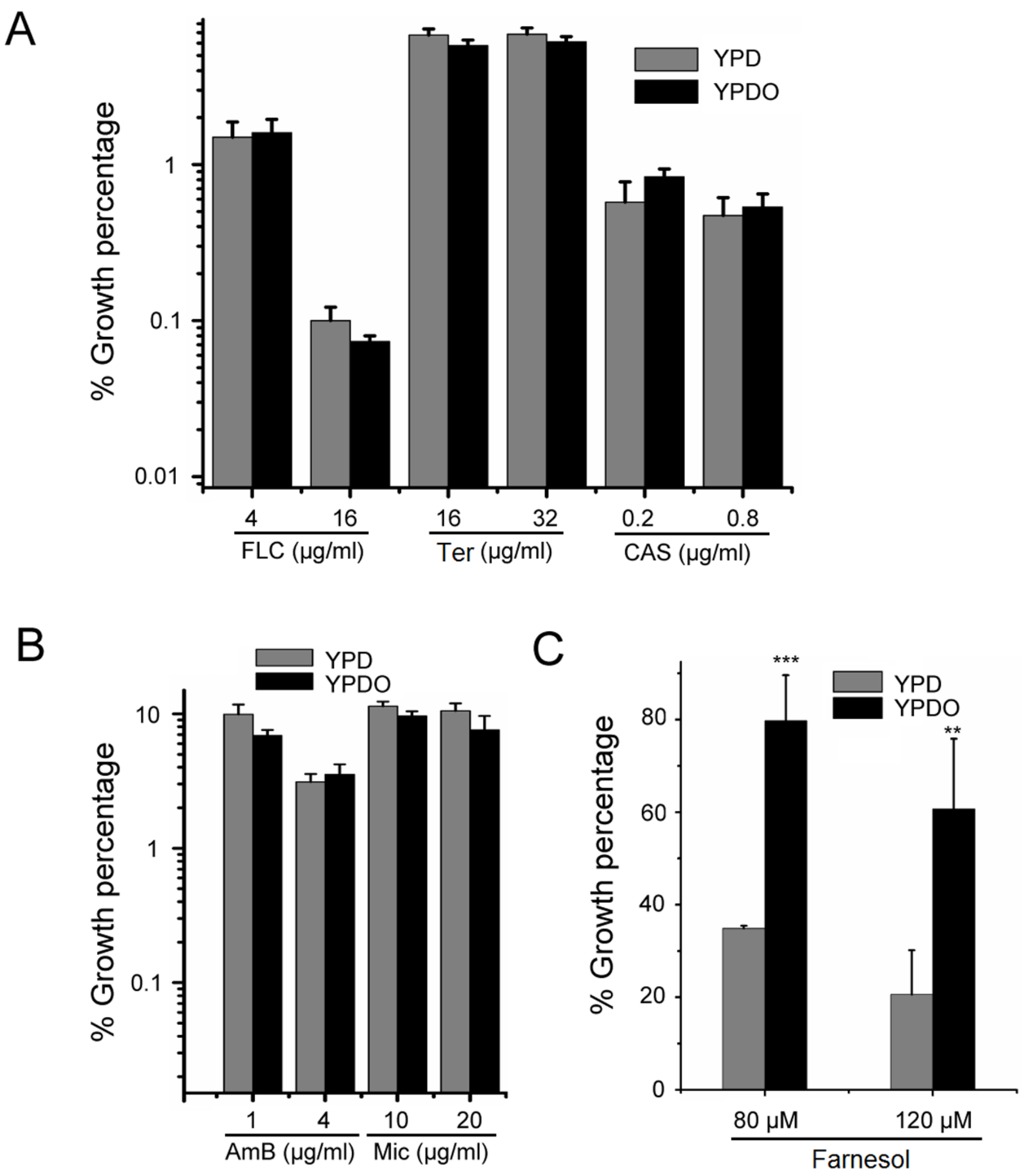

Supplementary Figure 10. The susceptibility of *C. albicans* to the tested antifungal agents when cultured in YPD or YPDO medium. *C. albicans* SC5314 was cultured in YPD or YPDO medium overnight. Cells were collected and treated with FLC for 12 h, CAS or Ter for 6 h (**A**), AmB, or Mic for 6 h (**B**) or farnesol for 6 h (**C**). The inhibitory effect for each treatment was evaluated based on the survival colony counting.

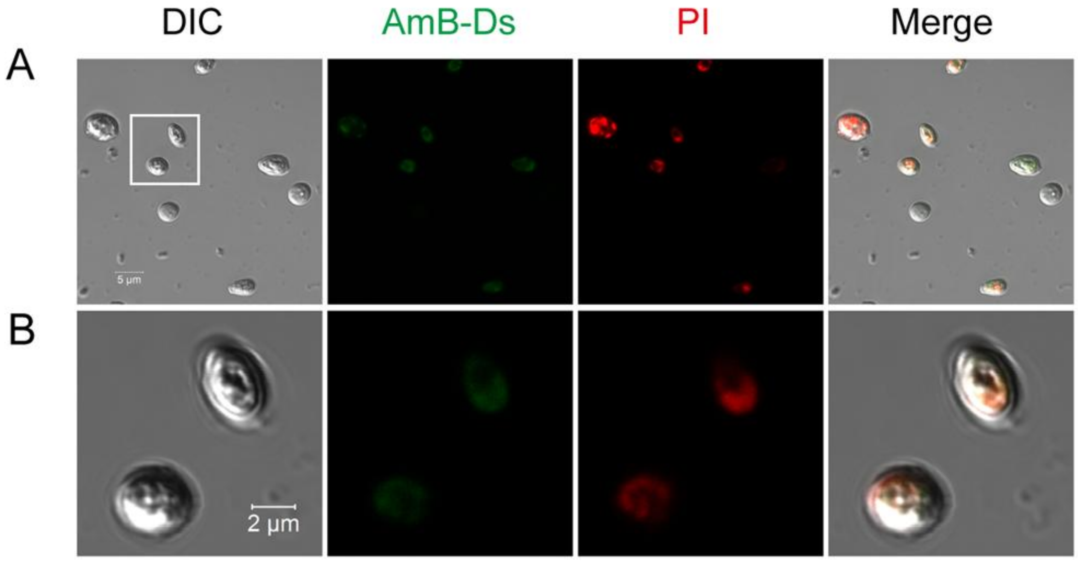

Supplementary Figure 11. The intracellular distribution of AmB indicated by its derivative AmB-Ds. *C. albicans* SC5314 was cultured in YPD and treated by AmB-Ds for 12 h at a final concentration of 30 μg/ml. Cells were collected and stained by PI for CLSM observation (**A**). The white rectangle area in **A** was taken by a closer examination (**B**).

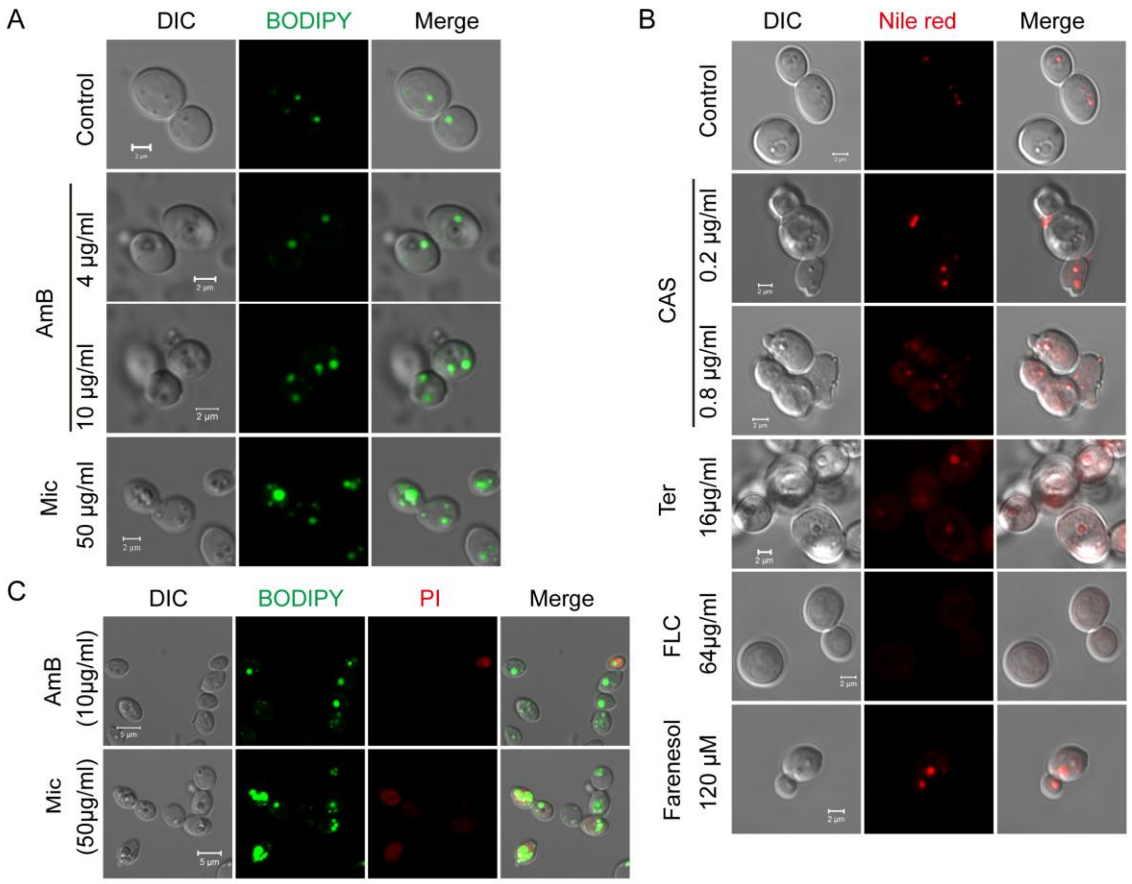

Supplementary Figure 12. The morphology of LDs in *C. albicans* treated with different antifungal agents. (**A**) The LD size of *C. albicans* was stimulated by AmB or Mic. *C. albicans* SC5314 was cultured in YPD and treated with AmB or Mic for 6 h. Cells were collected and stained with BODIPY for CLSM observation. (**B**) The morphology of LDs in *C. albicans* under the treatment of FLC, CAS, Ter or farnesol. *C. albicans* SC5314 was cultured in YPD and treated with FLC, CAS, Ter or farnesol for 6 h. Cells were collected and stained with Nile red for CLSM observation. (**C**) The viability of *C. albicans* cells under the treatment of AmB or Mic. AmB or Mic-treated cells were stained with BODIPY and PI for 30 min and subject to CLSM observation.

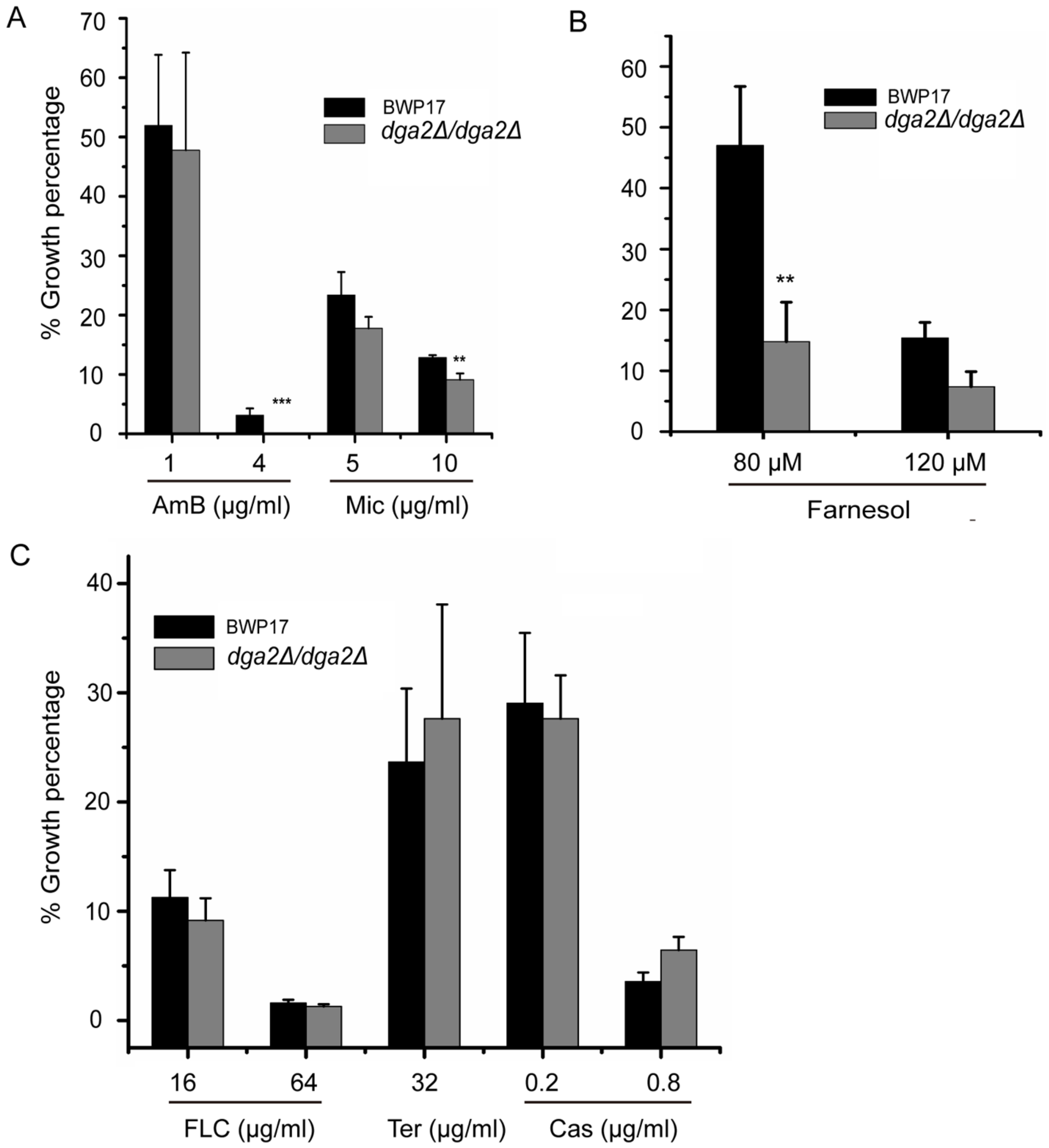

Supplementary Figure 13. The susceptibility of *C. albicans* mutant strains deficient in LDs formation to the antifungal agents.

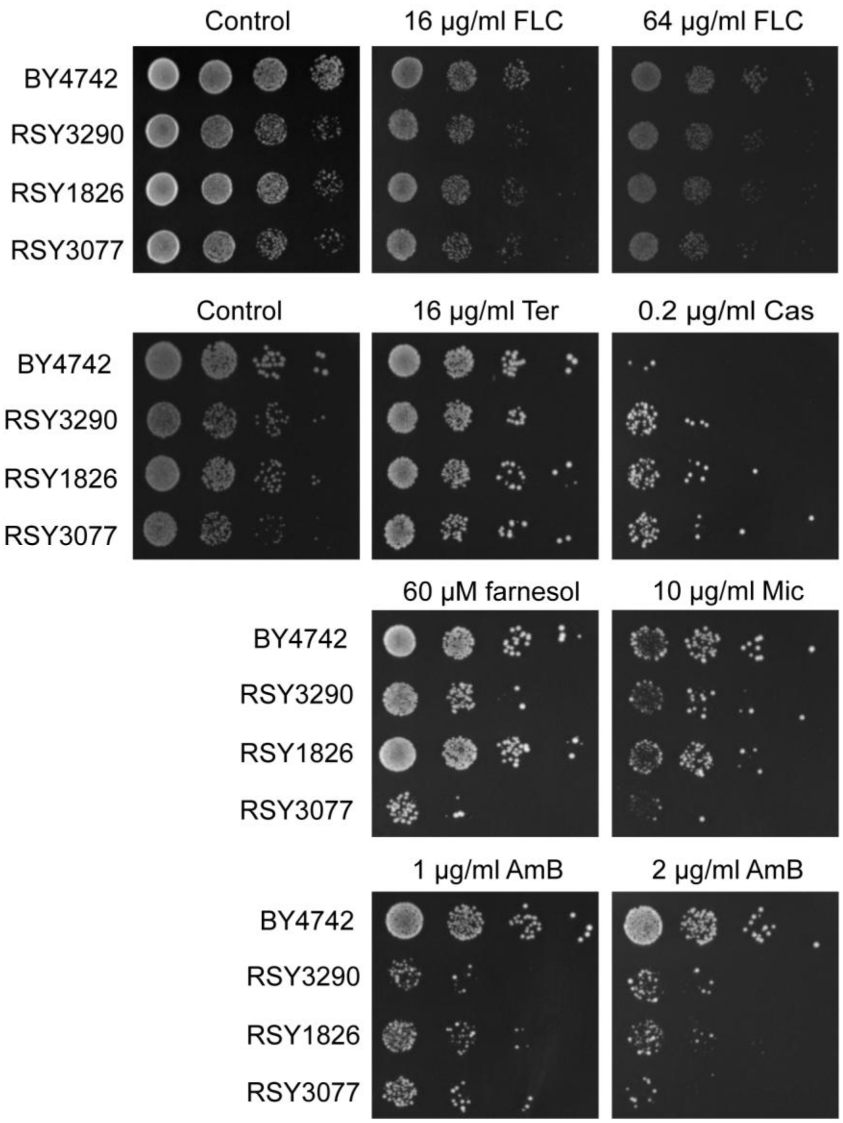

Supplementary Figure 14. The susceptibility of *S. cerevisiae* defective strains in LDs formation to antifungal agents assayed on YPD agar plates.

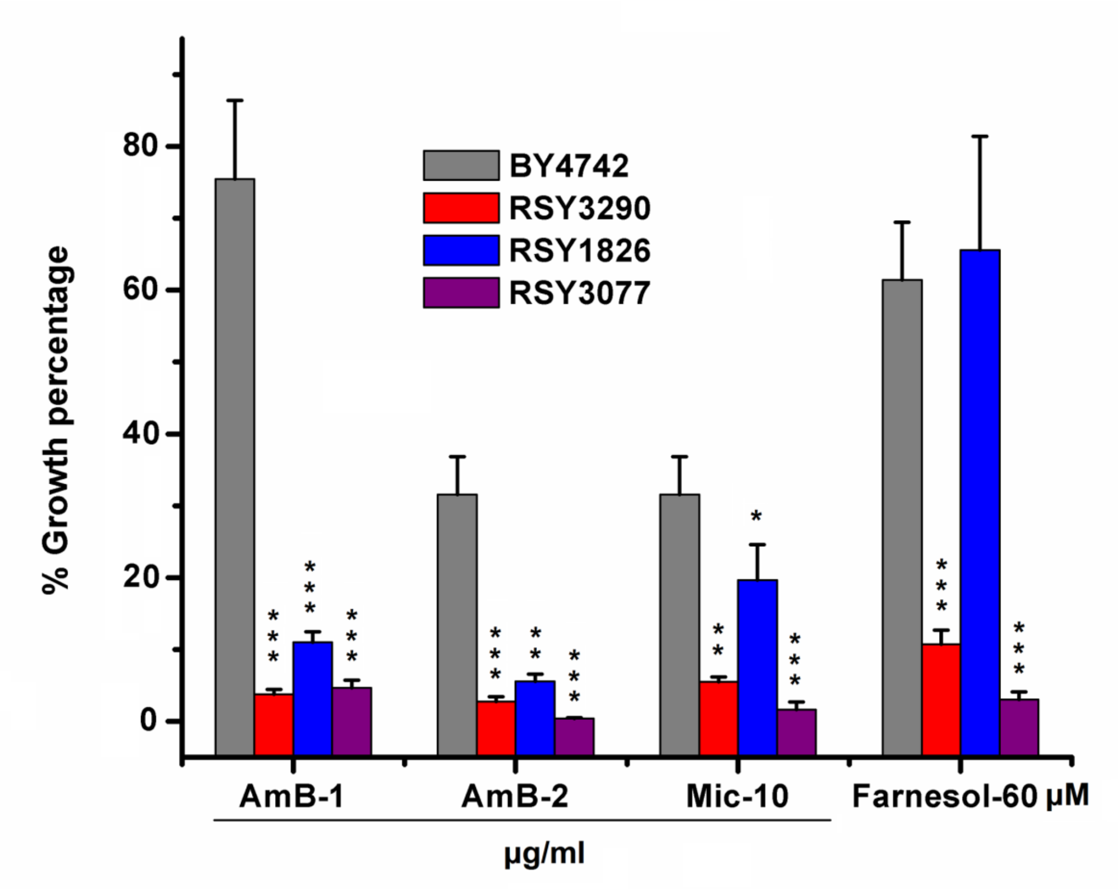

Supplementary Figure 15. The susceptibility of *S. cerevisiae* defective strains in LDs formation to antifungal agents of AmB, Mic and farnesol.

Supplementary References

1. Gillum, A. M., Tsay, E. Y. & Kirsch, D. R. Isolation of the *Candida albicans* gene for orotidine-5′-phosphate decarboxylase by complementation of *S. cerevisiae ura3* and *E. coli pyrF* mutations. *Mol. Gen. Genet.* **198**, 179-182.

2. Sun, L. *et al.* In vitro activities of retigeric acid B alone and in combination with azole antifungal agents against *Candida albicans*. *Antimicrob. Agents Chemother.* **53**, 1586-1591 (2009).

3. Wilson, R. B., Davis, D. & Mitchell, A. P. Rapid hypothesis testing with *Candida albicans* through gene disruption with short homology regions. *J. Bacteriol.* **181**, 1868-1874 (1999).

4. Jacquier, N. *et al.* Lipid droplets are functionally connected to the endoplasmic reticulum in *Saccharomyces cerevisiae*. *J. Cell. Sci.* **124**, 2424-2437 (2011).
